# On the Conditions Determining the Formation of Self-Crosslinking Chitosan Hydrogels with Carboxylic Acids

**DOI:** 10.3390/gels11050333

**Published:** 2025-04-29

**Authors:** Nils Münstermann, Oliver Weichold

**Affiliations:** Institute of Building Materials Research, RWTH Aachen University, Schinkelstraße 3, 52062 Aachen, Germany; muenstermann@ibac.rwth-aachen.de

**Keywords:** ionic hydrogel, carboxyalkylation, hydrogel, chitosan

## Abstract

The formation of self-crosslinking chitosan hydrogels using carboxylic acids has a number of limitations. Chitosan dissolves in oxalic, malonic, and succinic acids at a ratio of 1 amino group to 2 carboxyl groups into viscous solutions (G′ < G′′), but does not dissolve with lower amounts of the acid. Mixing chitosan hydrochloride with disodium carboxylates does not afford gels, but only a coacervate in the case of disodium oxalate, which dissolves upon dialysis. In the homologous series of *N*-carboxyalkyl derivatives (alkyl = methyl, ethyl, propyl), all members form gels (G′ > G′′). At approx. 50% of substitution, the storage modulus increases from 40 Pa (methyl) to 30,000 Pa (propyl) indicating the increasing strength of intermolecular interactions with the increasing length of the alkyl spacer. This could indicate that a sufficiently long spacer is required to properly connect the chitosan helices. *N*-succinyl chitosan, where the spacer is attached to the backbone as an amide, also forms polymer gels across all degrees of *N*-acylation. When compared to *N*-carboxypropyl chitosan, the latter forms significantly stiffer gels that swell less. This indicates that one covalent bond, a sufficient length, and the conformational flexibility of the spacer are important for gelation.

## 1. Introduction

Chitosan is a biopolymer with a wide range of molecular sizes, usually ranging between 1 and 600 kDa. According to current knowledge, it occurs in nature only in the cell walls of a few species of fungi and is therefore obtained by a chemical conversion of chitin on a large scale [1]. The structural difference between chitin and chitosan lies in the quantitative ratio of acetylglucosamine to glucosamine repeat units. If the proportion of glucosamine exceeds 50%, the polymer is referred to as chitosan [1]. This ratio, usually expressed as the degree of deacetylation (DA), strongly influences the solubility in water, as the free amino groups are easily protonated by e. g. acetic, lactic, propionic, or citric acid to form water-soluble salts [1]. Once dissolved, chitosan can be chemically modified using the hydroxy and amino groups to alter the solubility of chitosan in both acidic and alkaline environments, as well as to improve the biodegradability, swelling behaviour and biocompatibility.

The cross-linking of water-soluble polymers results in the formation of hydrogels. These are an important class of materials especially for biomedical applications as a large number of biological tissues consist of water-swollen hydrogels [2,3]. Due to toxicological issues, hydrogels from bio-based polymers are of particular interest. The preparation of hydrogels from covalently cross-linked chitosan only requires chitosan and a cross-linking agent in a suitable solvent, preferably water. The most common cross-linking reagents for chitosan to date are dialdehydes such as glyoxal [4] and glutardialdehyde [5]. The advantage of cross-linking with dialdehydes is that a direct reaction in aqueous media is possible under mild conditions and without the addition of auxiliary molecules such as reducing agents [6]. A disadvantage is the toxicity of dialdehydes [7]. Glutardialdehyde, for example, is considered neurotoxic [8] and glyoxal is known to be a mutagen [9]. Even with careful purification of the hydrogels, the presence of free, unreacted dialdehydes in hydrogels cannot be completely ruled out [10]. Chitosan can also be covalently crosslinked with dicarboxylic acids using 1-ethyl-3-(3-dimethylaminopropyl)carbodiimide (EDC) and *N*-hydroxysuccinimide (NHS) as coupling agents [11,12,13]. However, additional toxic and expensive cross-linking reagents are required, which would also have to be carefully removed after synthesis. Almost at the other end of the bond-strength scale are hydrogen bonds and the hydroxy groups of chitosan have already been used to form non-covalent gels [14]. However, the disadvantage is the potential lack of mechanical stability and the risk of dissolution upon swelling [10]. Chitosan offers a third option without the necessity of extensive chemical modification: ionic cross-linking. While multifunctional molecules are required as cross-linkers for covalent cross-linking, multivalent counterions are required as crosslinkers for ionic cross-linking in order to connect the polymer chains [10]. Similarly to covalently cross-linked hydrogels, the cross-linking density is the main parameter that influences the important properties of ionically cross-linked hydrogels, such as mechanical strength, swelling behaviour, and the exchange/release properties [15]

**Figure 1:**
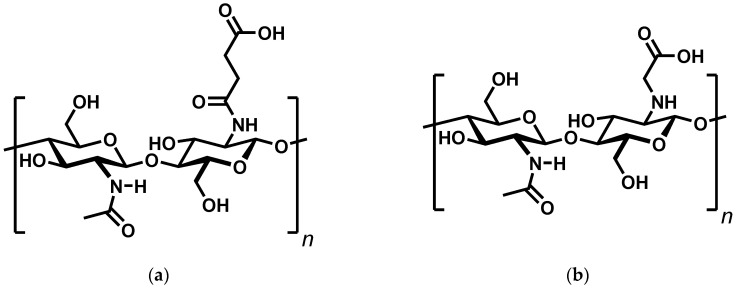
Structure of *N*-Succinyl chitosan (**a**) and *N*-Carboxymethylchitosan (**b**).

Continuing our previous research on the interaction of chitosan and monoprotic carboxylic acids [16], we were interested in preparing chitosan hydrogels by acid–base interactions using simple, low molecular weight carboxylic acids. The advantages of such physically bound hydrogels are their environmental friendliness and the potential for self-healing. A literature search led to two structurally similar but very different compounds in terms of their properties: *N*-succinyl chitosan [17] and *N*-carboxymethylchitosan [18] (Figure 1).

In water, the former was found to be a gel, while the latter was reported to form viscous solutions. The question thus arose, why two compounds with similar structural functionality, equal type of interaction, and similar pK_A_ values behave so differently. To answer this question, this article examines the conditions for ionic cross-linking of chitosan using a set of mono- and diprotic carboxylic-acids with two to four carbon atoms.

## 2. Results and Discussion

**Figure 2:**
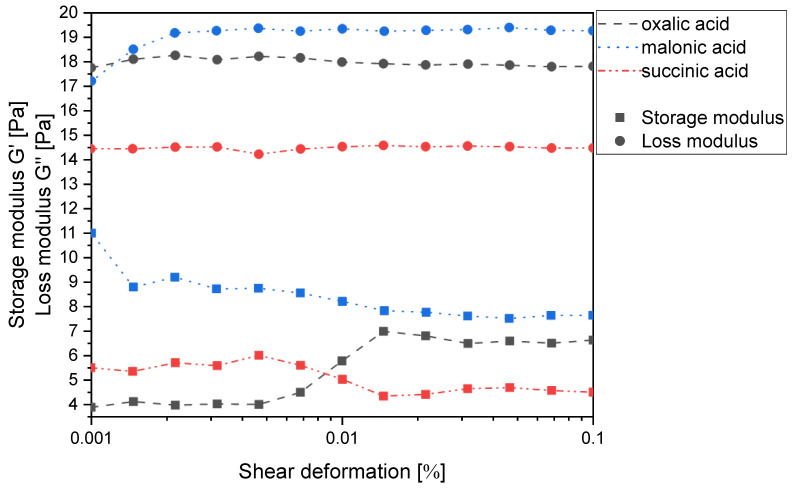
Storage modulus G′ and loss modulus G′′ of the chitosan salts of oxalic, malonic and succinic acid (1 wt% in water based on chitosan) as a function of the shear deformation γ.

Chitosan can be easily dissolved in dilute acids, whereby the corresponding salts are formed by protonation of the amino group. According to our previous findings, the prerequisite for this and for avoiding depolymerization of the chitosan backbone is adding 0.95 to 0.99 equiv. (relative to the number of free amino groups) of a sufficiently strong and water-soluble carboxylic acid with a pK_A_ < 5.0 [16]. In the homologous series of monocarboxylic acids, only the first four (formic to butyric acid) meet this requirement and solubilize chitosan. Building on this idea, the simplest way to produce chitosan gels without reactive cross-linkers, therefore, appeared to be the use of dicarboxylic acids. Not surprisingly, the homologous series of dicarboxylic acids (oxalic-, malonic- and succinic acid) easily solubilize chitosan in water when used in an equimolar ratio (1:2 ratio of free amino and carboxyl groups), since all three pK_A1_ values meet the above requirement. Under these conditions, the number of carboxylic acid groups is twice as high as that of the free amino groups, so the second interaction that could cause cross-linking of the chitosan chains can be either a hydrogen bond or dipole-charge interaction. However, all mixtures were clearly identified as viscous polymer solutions and not as gels, as the loss moduli exceeded the corresponding storage moduli in all cases (Figure 2). This was not surprising, as we have already shown that chitosan does not form a gel with itaconic acid, which is an α,β-unsaturated derivative of succinic acid, either [19].

Chitosan could not be dissolved using 0.5 equiv. of the dicarboxylic acids (1:1 ratio of free amino and carboxyl groups). For malonic and succinic acid, the pK_A2_ values are >5 so this result corresponds to our expectations. In the case of oxalic acid, the pK_A_ values are 1.23 and 4.19, which suggests that both carboxyl groups are able to protonate the amino groups on chitosan. It should be noted that the addition of finely powdered chitosan to equimolar aqueous solutions of the monosodium salts of the three acids (1:1:1 ratio of free amino, carboxyl, and carboxylate groups) only forms solutions in the case of oxalic acid. This corroborates the pK_A_ criterion cited above. Thus, in accordance with the pK_A_ values of oxalic acid, mixing 0.5 equiv. oxalic acid with chitosan should form an ionically self-crosslinking chitosan gel. The reason why neither dissolution of the chitosan nor gel formation is observed under these conditions is not entirely clear. In view of the findings outlined so far, one explanation could be that there is indeed a thin layer of gel forming at the solid–liquid interface. However, this would be characterised by a high polymer volume fraction. In such a case, swelling is described by a combined diffusion-relaxation model rather than pure Fickian behaviour [20]. Given the many strong interactions in solid chitosan [21], chain relaxation can be expected to be slow and to dominate the swelling behaviour. This delays further ingress in such a way that macroscopically no reaction is observed.

Trying to force the systems to gel by reacting chitosan hydrochloride with the disodium salts of the dicarboxylic acid (1:1 ratio of ammonium ions and carboxylates) as described earlier [19], only led to a second, cloudy phase in the case of disodium oxalate. However, when dialysing this phase against double distilled water, it dissolved into a clear polymer solution. The formation of the second phase in the system chitosan hydrochloride-disodium oxalate seems to be, therefore, coacervation, driven by the presence of 6.2 mol/L NaCl in the mixture rather than gelation. For the sake of completeness, it should be mentioned that citric acid behaves very similarly to oxalic acid, as two of the three pK_A_ values are below 5. This means chitosan dissolves in citric acid solutions at a ratio of 1 free amino group to 1 molecule of acid. It does not dissolve in solutions of disodium citrate, since the pK_A_ value of the remaining carboxyl group is >5. Chitosan hydrochloride forms gels with disodium and trisodium citrate in 1:1 ratios of ammonium ions to carboxylates, which dissolve when dialysed.

**Figure 3:**
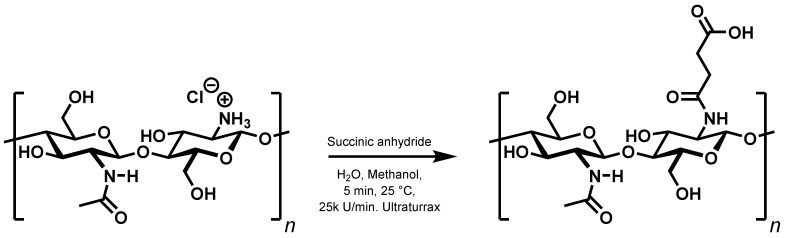
Modified synthesis of *N*-succinyl chitosan.

The results above seem to indicate that persistent, purely ionic gels cannot be prepared from chitosan and carboxylic acids. The next consequent step is to replace one ionic interaction with a covalent bond. There are two simple ways to accomplish this, either by reacting dicarboxylic acids with the free amino groups to form monoamides, or to alkylate the free amino groups using ω-halocarboxylic acids. As outlined in the introduction, the first method has previously been used to synthesise *N*-succinyl chitosan from chitosan hydrochloride and succinic anhydride [17] (Figure 3).

**Figure 4:**
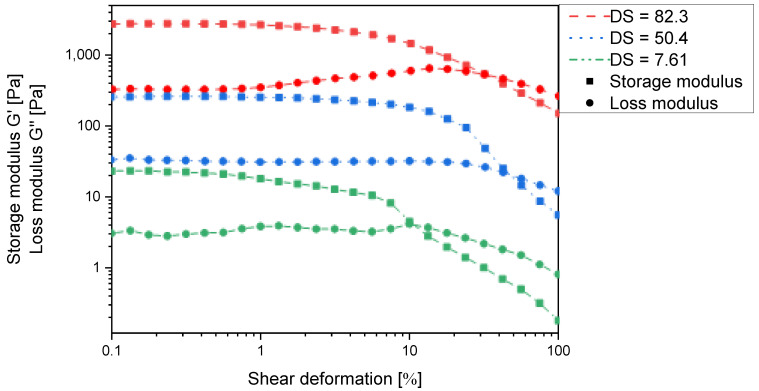
Storage modulus G′ and loss modulus G′′ of *N*-succinyl chitosan hydrogels in the maximum swollen state according Figure 5 as a function of the shear deformation γ and the degree of *N*-substitution.

In order to investigate the extent of interactions between the chains, a set of *N*-succinyl derivatives with a degree of substitution ranging from 8% to 100% were synthesised (Supplementary Table S1). All of these derivatives form polymer gels in water as indicated by the storage modulus exceeding the loss modulus in all cases (Figure 4). This implies that strong intermolecular interactions occur between the individual chitosan chains and corroborates the intended ionic self-cross-linking effect.

**Figure 5:**
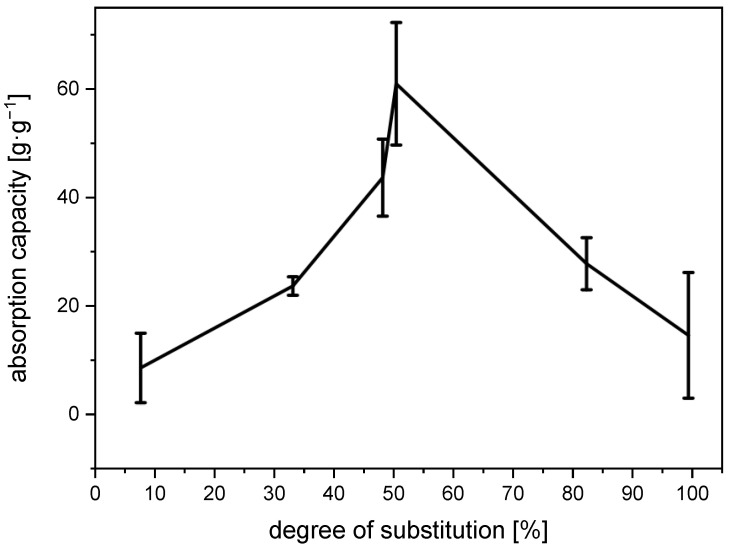
Water absorption capacity of *N*-succinyl chitosan gels depending on the degree of substitution.

The water-absorption capacity of the *N*-succinyl chitosan gels initially rises to a peak of 61 g∙g^−1^ at a degree of substitution of approx. 50% and then drops to approx. 15 g∙g^−1^ at complete substitution (Figure 5). The initial increase in the absorption capacity is due to an increase in the number of charges and with it the number of CO_2_^−^⋯H_3_N^+^ interactions at higher degrees of substitution. Likewise, the subsequent drop in absorption capacity could be caused by a decreasing number of charge-charge interactions. Beyond 50% of substitution, every free amino group that is converted to the corresponding amide can no longer be protonated.

**Figure 6:**
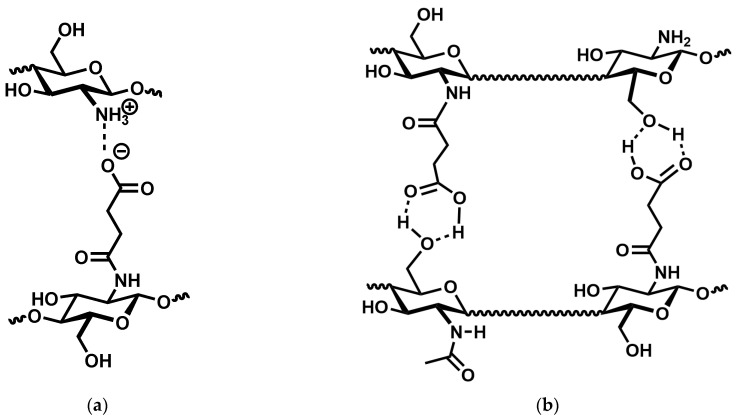
Possible ionic interactions between the amin o groups of chitosan and the carboxyl groups of *N*-succinyl chitosan (**a**) and hydrogen bonding between the hydroxyl group of chitosan and the acid group of *N*-succinyl chitosan. (**b**). Dashed lines indicate non-covalent interactions, wavy lines an unspecified continuation of the chitosan backbone.

However, Figure 4 clearly indicates that the storage modulus continuously increases with increasing degree of substitution and is approx. one order of magnitude higher at 82% substitution than at 50% substitution. The latter can only be explained by an increase in cross-linking density, which would also lead to a reduced absorption capacity. It thus appears that in *N*-succinyl chitosan, there are two dominant cross-linking mechanisms operating: charge–charge interaction and hydrogen bonding (Figure 6). However, the present results do not allow to decide whether the latter are COOH⋯OH, COOH⋯HNCO, or a mixture of both.

**Figure 7:**
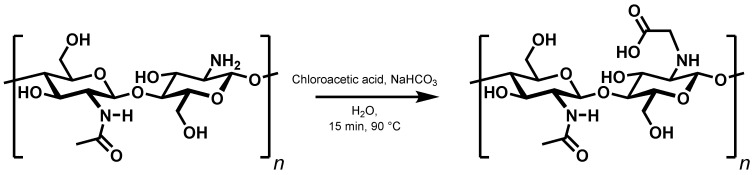
Synthesis of *N*-carboxymethylchitosan.

The second way of introducing a covalent bond, *N*-alkylation, has previously been used to synthesise *N*-carboxymethylchitosan from chitosan and 2-chloroacetic acid (Figure 7) [18]. Here, too, the extent of interactions between the chains was checked by investigating a set of derivatives with a degree of substitution ranging from 10% to 99% (Supplementary Table S2).

**Figure 8:**
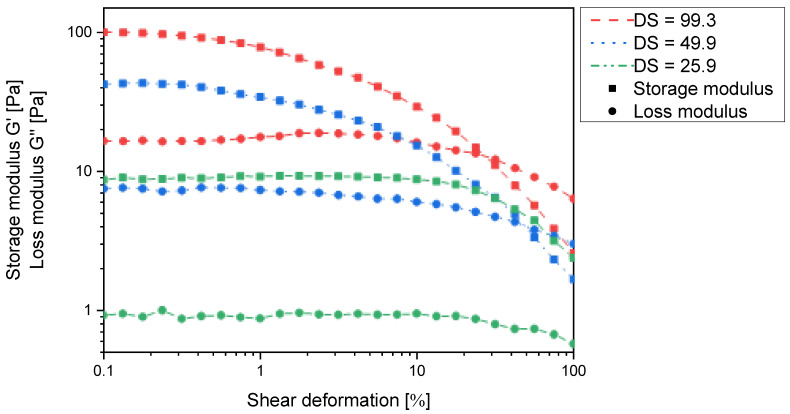
Storage modulus G′ and loss modulus G′′ of *N*-carboxymethylchitosan hydrogels (1 wt% in water) as a function of the shear deformation γ and the degree of *N*-substitution.

Previous reports claimed *N*-carboxymethylchitosan with up to 47% *N*-substitution to be water soluble [18,22,23]. A close examination of the rheological data in Figure 8 clearly shows that the derivatives actually form polymer gels over the entire range of substitution degrees, as the storage moduli exceed the loss moduli in all cases. The observed discrepancy to the literature findings could be due to differences in the molecular weight of the chitosan used. The absolute values of the storage moduli and particularly those of the loss moduli (<20 Pa) are quite small so that the viscous properties are still very pronounced, and the gels cannot be isolated to analyse e.g., their swelling behaviour. In fact, when diluted, the gels dissolve. The viscous properties are expected to be even more pronounced when using chitosan of lower molecular weight. Nevertheless, the storage moduli increase with increasing degree of substitution, and it is most likely that a similar argument to that of *N*-succinyl chitosan can be made.

**Figure 9:**
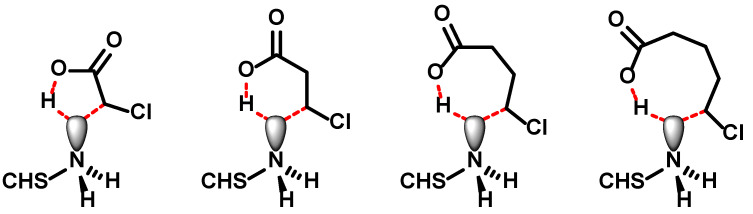
Potential transition states during the intramolecular alkylation of chitosan using ω-halocarboxylic acid. The dashed red lines indicate non-covalent interactions.

Both *N-*succinyl chitosan and *N*-carboxymethylchitosan are capable of forming charge–charge and charge–dipole interactions as well as hydrogen bonds. In addition, the pK_A_ value of ω-substituted carboxylic acids increases with increasing number of methylene groups. That is, *N*-carboxymethylchitosan should be more acidic than *N-*succinyl chitosan and should, therefore, form stronger interactions, but the opposite is observed. The striking differences between the gel properties could, thus, originate in two structural differences: the additional amide bond in *N*-succinyl chitosan and the chain length. *N*-Malonylchitosan and *N*-oxalylchitosan are not available for analysing the effect of the chain length, since the required anhydrides are unknown. Instead, *N*-carboxyethylchitosan [24] and *N*-carboxypropylchitosan were selected to complete a systematic set of *N*-carboxyalkyl derivatives with two to four carbon atoms and to obtain an alkylated equivalent of *N-*succinyl chitosan in order to compare their properties. The preparation of the higher *N*-carboxyalkyl derivatives follows the procedure described for *N*-carboxymethylchitosan. By increasing the amount of ω-halocarboxylic acid, higher degrees of substitution were obtained. However, the maximum degree of substitution decreased with increasing chain length of the acid from >99% for *N*-carboxymethylchitosan to 56.4% for *N*-carboxyethylchitosan and to 46.4% for *N*-carboxypropylchitosan even at an exorbitant excess of reagent. The next in line, *N*-carboxybutylchitosan, could not synthesised. At this point, it is important to remember that in order to solubilize chitosan, the free amino groups need to be protonated. Hence, the nitrogen lone pair is not available for nucleophilic substitution. It can be envisioned that the interaction of the carboxylate with the ammonium ion draws the halogenated chain end into proximity and in a concerted reaction the proton is transferred back to the carboxylate, releasing the lone pair, which simultaneously attacks the ω-carbon atom to close the C–C-bond (Figure 9). While the transition state for ω-chloroacetic, -propionic and -butyric acid adopts a 5-, 6-, or 7-membererd ring, which are energetically feasible, the 5-chloropentanoic acid—required for the preparation of *N*-carboxybutylchitosan—would need to go through an 8-membered ring, which is highly unfavourable. This is corroborated by two observations: (i) 4-chloropentanoic acid can be used to prepare the *N*-(1-methyl)carboxypropyl chitosan [25], which would also proceed through a 7-membererd ring, and (ii) that interchain alkylation does not occur as the simultaneous COO^−^⋯H_3_N^+^ interaction is missing.

**Figure 10:**
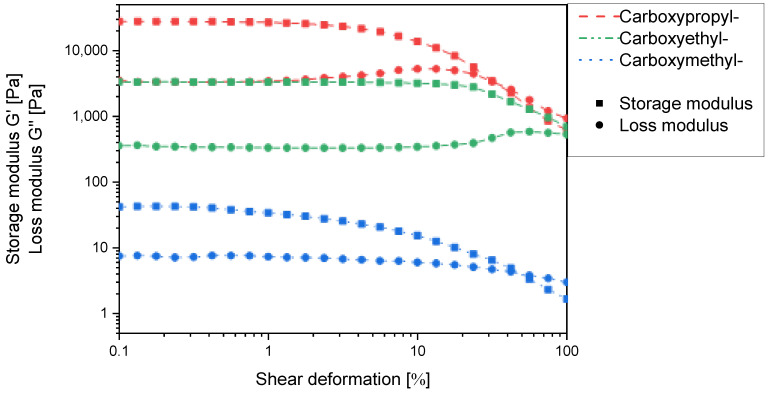
Storage modulus G′ and loss modulus G′′ of *N*-carboxymethyl-, *N*-carboxyethyl-, *N*-carboxypropylchitosan hydrogels in the maximum swollen state as a function of the shear deformation γ at the degree of ~50% *N*-substitution.

Due to the experimental constraints, the set of *N*-carboxyalkyl chitosans was compared using the derivatives with approx. 50% substitution. Here, the storage and loss moduli increase with increasing chain length in such a way that *N*-carboxyethyl and *N*-carboxypropyl chitosan form macroscopically stable gels (Figure 10). It thus appears that for one chitosan helix to interact with a second one via a non-covalent spacer, a certain distance and/or conformational flexibility is required. When comparing *N*-carboxypropylchitosan (46.4% substitution) with *N*-succinyl chitosan (48.15% substitution), it is noticeable that the former has a lower swelling capacity (38.4 g∙g^−1^ vs. 43.7 g∙g^−1^), but the storage modulus is approx. two orders of magnitude higher. This indicates that the *N*-carboxypropyl chitosan forms much stronger intermolecular interactions than the *N*-succinyl chitosan. As the only structural difference between these two compounds is the amide function, and this is a rigid structural element, the contribution of conformational flexibility to the intermolecular interaction must not be underestimated.

## 3. Conclusions

Purely ionic gels containing only chitosan and polyprotic carboxylic acids cannot be made by direct protonation. At best, these mixtures form coacervates when, for example, solutions of chitosan hydrochloride and the polycarboxylate are combined, provided that at least two of the carboxyl groups have pK_A_ values below 5 and an additional electrolyte such as the original counterions is present in the medium. These coacervates dissolve when dialysed indicating that the process is driven by the decreasing solubility of the polyion complex with increasing ionic strength. Instead, the preparation of ionic gels using carboxylate anions requires attaching a sufficiently long and flexible spacer. For *N*-carboxyalkyl derivatives, a spacer of one methylene group (*N*-carboxymethyl) leads to extremely weak gels, while three methylene groups (*N*-carboxypropyl) afford very firm gels. A spacer of similar length but attached as amide (*N*-succinyl chitosan) provides significantly softer gels with a higher swelling ratio, which indicates the importance of spacer flexibility. A comparison with literature results suggests that the length of the chitosan chain and the degree of deacetylation also play a role, and shorter types with fewer free amino groups than used here (${\bar{M}}_{v}$ = 1072 kDa, 88% deacetylation) will show a reduced stiffness and increased swelling ratio. Thus, the properties of these ionic chitosan gels can be controlled by the chain length and degree of deacetylation of the chitosan, the degree of substitution, as well as the length and flexibility of the spacer. The conditions described above for the production of environmentally friendly, toxicologically safe, and durable gels and the possibility of further functionalising the chitosan backbone could help to customise the next stage of biomedical hydrogels.

## 4. Materials and Methods

Chitosan flakes (degree of deacetylation 88.01%, ${\bar{M}}_{v}$ = 1072 kDa) were purchased from TCI Deutschland GmbH, Eschborn, Germany and lyophilised to a constant weight. The degree of deacetylation was determined by conductometric titration according to dos Santos et al. [26] using a GMH 3430 conductometer from Senseca Germany GmbH, Regenstauf, Germany. For this, 50 mg chitosan was dissolved in 20 mL of 0.05 M hydrochloric acid, stirring constantly at room temperature. A total of 0.1 M sodium hydroxide solution was used for the titration. All titrations were carried out in duplicate. The intrinsic viscosity was measured with a Xylem 501 13/Ic Ubbelohde viscometer (*K* = 0.03059 mm^2^/s^2^) in 0.25 M acetic acid and sodium acetate buffer solution with a pH value of 4.34 at 20 ± 0.05 °C. A series of seven samples in the range of 0.0–3.0 mg/mL in 0.5 mg/mL increments were measured. Before the measurements, the solutions were passed through a 1 μm syringe filter. All measurements were carried out in quadruplicate. The viscosity average molecular mass was calculated using the Mark–Houwink equation, following the procedure and applying the *k* and α values reported by Wang et al. [27].

Milling of the chitosan was performed on a Fritsch Pulverisette 14 Premium Line from Fritsch GmbH, Idar-Oberstein, Germany. For this purpose, the cutting insert with a 0.2 mm hole size was selected.

As a high-performance dispersing unit, the T 25 digital ULTRA-TURRAX^®^ from IKA, Staufen im Breisgau, Germany, was used to disperse the solutions, which in some cases were highly viscous. The solutions were sheared at the maximum speed of 25,000 rpm.

Infrared spectra were recorded on a Spectrum Two with a UATR attachment from Perkin Elmer, Waltham, MA, USA. Twenty-four scans were acquired in a measurement range of 400–4000 cm^−1^. Sample drying was performed on a Laborgene Scanvar Coolsafe freeze dryer (Supplementary Figures S1 and S2).

For the preparation of chitosan salt solutions, 100 mg of chitosan was placed in 10 mL of an aqueous solution containing 1 eq. (relative to the amount of free amino groups of chitosan) of a carboxylic acid such as oxalic, malonic, or succinic acid. The chitosan was completely dissolved under vigorous stirring with a high-performance disperser, leading to a 1 wt.% (based on pure chitosan) solution of the chitosan salt. For dissolution tests, the mono- or disodium salts of the acids were added to a 1 wt.% solution of chitosan or chitosan hydrochloride, prepared from chitosan a 1 eq. hydrochloric acid as described above.

Further experimental details and the procedures for synthesising the chitosan derivatives can be found in the Supplementary Materials.

## Data Availability

Data are available upon reasonable request.

## Supplementary Materials

The following supporting information can be downloaded at: https://www.mdpi.com/article/10.3390/gels11050333/s1, Figure S1: IR-spectra of *N*-Succinylchitosan at different degrees of substitution; Figure S2: IR-spectra of *N*-carboxyalkyl chitosan; Table S1: Absorption capacity of *N*-Succinylchitosan depending on the degree of substitution; Table S2: *N*-carboxymethylchitosan with different degrees of substitution; Table S3: Absorption capacity of *N*-Carboxyethylchitosan depending on the degree of substitution; Table S4: Absorption capacity of *N*-Carboxypropylchitosan depending on the degree of substitution. References [17,18,26] are cited in the Supplementary Materials.

## Abbreviations

The following abbreviations are used in this manuscript:

DA	Degree of deacetylation
EDC	1-ethyl-3-(3-dimethylaminopropyl)carbodiimide
NHS	*N*-hydroxysuccinimide
