# Supplementary material for: On the Conditions Determining the Formation of Self-Crosslinking Chitosan Hydrogels with Carboxylic Acids
Source: Gels. 2025 Apr 29;11(5):333. doi: 10.3390/gels11050333 (PMC12111127; doi:10.3390/gels11050333)
Supplement: Supplementary file 1 [file gels-11-00333-s001.zip › gels-3577306-supplementary.pdf]

# Supporting Information to

# On the conditions determining the formation of self-crosslinking chitosan hydrogels with carboxylic acids

Nils Münstermann, Oliver Weichold

Institute of Building Materials Research, RWTH Aachen University, Schinkelstraße 3, 52062 Aachen, Germany

Number of pages: 5

Number of tables: 4

Number of figures: 2

## Materials and Methods

Acetone, hydrochloric acid and sodium hydrogen carbonate were purchased from VWR International. Succinic anhydride and 4-chlorobutyric acid were purchased by Sigma-Aldrich. Acetic acid (1 M), ethanol, methanol, monochloroacetic acid and sodium hydroxide solution (1 M) were purchased by Merck. Oxalic acid was purchased by Acros organics. If not mentioned otherwise, these were used without further purification.

**Rheology measurements** were performed on an Anton Paar MCR 102 rheometer. A plate-cone geometry (1-CP25/SS, Ø = 25 mm) with a diameter of 25 mm made of stainless steel was used. 0.5 mL of the samples were measured with a measuring gap of 1 mm at a temperature of 20 °C.

**Elemental analyses** for the determination of the degree of substitution of the chitosan derivatives were performed using a Vario EL Cube (Elementar Analysensysteme GmbH, Langenselbold, Germany). The DA was calculated from the mass ratio between the detected carbon and nitrogen atoms $w_{C/N}$, following the procedure of dos Santos et al. (dos Santos, Caroni, Pereira, da Silva, & Fonseca, 2009).

**Absorption properties** were determined with tea bag test. The polyester filter bags were purchased from Rosin tech labs (San Francisco, USA). They are 4.4 cm × 20 cm in size and have a mesh size of 90 µm. To determine the absorption kinetics in doubly distilled water, 50 mg of sample were placed in a moistened and pre-weighed tea bag. The bag was then placed in 200 mL of doubly distilled water. After defined time intervals (5, 15, 30, 60, 120, 240, 1440 min.), the bag was removed, blotted three times per side on a paper and then weighed. The absorption capacity is calculated according to the following equation.

$$AC = \frac{m_3 - m_2 - m_1}{m_1} \quad (2)$$

AC: absorption capacity [$g \cdot g^{-1}$];

$m_3$: mass dried SAPs [g];

$m_2$: mass moistened polyester tea bags [g];

$m_1$: mass of the polyester tea bag filled with swollen SAPs [g];

**Preparation of *N*-succinylchitosan**

The *N*-succinyl chitosan was prepared following the procedure of Yamaguchi *et al.* (Yamaguchi, Arai, Itoh, & Hirano, 1981) with some modifications: 2 g of chitosan were placed in 90 mL of water. 10.591 mL 1 м hydrochloric acid 1 equiv. relative to the amount of free amino groups) was added under vigorous agitation using a high-performance disperser. After the chitosan had dissolved completely, the solution was diluted with 150 mL of methanol. A solution of the desired amount of succinic anhydride (0.5 to 3 equiv. relative to the amount of free amino groups of the used chitosan) in 50 mL of acetone was added to the chitosan hydrochloride solution with vigorous agitation using a high-performance disperser at 25000 rpm for 5 minutes while cooling with an ice bath. Subsequently, the methanol and the acetone were removed using a rotary evaporator. After adding 200 mL of water, the pH was adjusted to pH = 7 using ammonia. To separate the salts, the solution was dialyzed against double-distilled water for 3 days, changing the water every day. The purified hydrogel was concentrated to a volume of approx. 100 mL on the rotary evaporator and finally dried by freeze drying. *N*-succinylchitosan was obtained as a colourless solid foam. The degree of

substitution was determined by elemental analysis from the mass ratio of carbon to nitrogen $w_{C/N}$.

***Table S-1.*** *Absorption capacity of N-Succinylchitosan depending on the degree of substitution.*

| Equivalents of succinic anhydride [-] | Degree of substitution [%] | Absorption capacity [g·g$^{-1}$] |
|---|---|---|
| 0.1 | 7.6 ± 0.3 | 8.6 ± 6.4 |
| 0.5 | 33.1 ± 1.1 | 23.7 ± 1.7 |
| 1 | 48.2 ± 0.6 | 43.7 ± 7.1 |
| 1 | 50.4 ± 1.9 | 61.0 ± 21.3 |
| 2 | 82.3 ± 2.7 | 27.8 ± 4.8 |
| 3 | 99.4 ± 1.8 | 14.6 ± 11.6 |

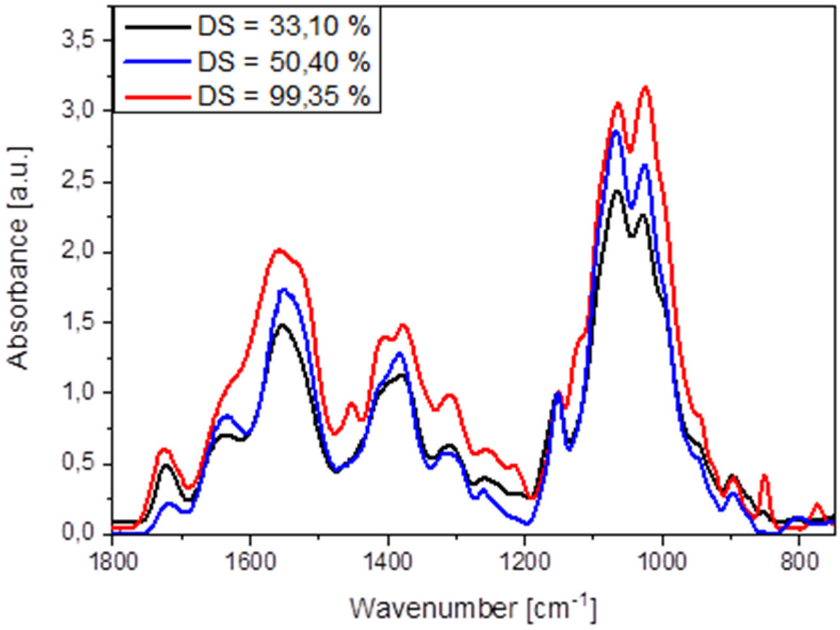


**Figure S-1:** IR-spectra of *N*-Succinylchitosan at different degrees of substitution.

**Preparation of *N*-carboxyalkyl chitosan**

*N*-carboxyalkyl chitosan was prepared following the procedure of An *et al.* with some modifications (An, Thien, Dong, & Dung, 2009). 2 g of chitosan were placed in 200 mL of water. The desired amount of an ω-chlorocarboxylic acid (0.5 to 3 equiv. relative to the amount of free amino groups of the used chitosan) was added to the solution under vigorous agitation using a high-performance disperser. Stirring was continued until the chitosan was completely

dissolved. The pH was adjusted to a value between 8 and 8.5 by carefully adding sodium carbonate solution (10 wt% in water) under vigorous stirring. The solution became opaque due to precipitating chitosan. The solution was heated to 90 °C for exactly 15 minutes and then slowly cooled to room temperature. The product was precipitated by lowering the pH by addition of hydrochloric acid (0.1 м) to approx. pH 6. The solution was dialysed for three days in doubly distilled water changing the water every day. The product was obtained as a colourless foam by drying on the freeze dryer. The degree of substitution of was determined by elemental analysis from the mass ratio of carbon to nitrogen $w_{C/N}$.

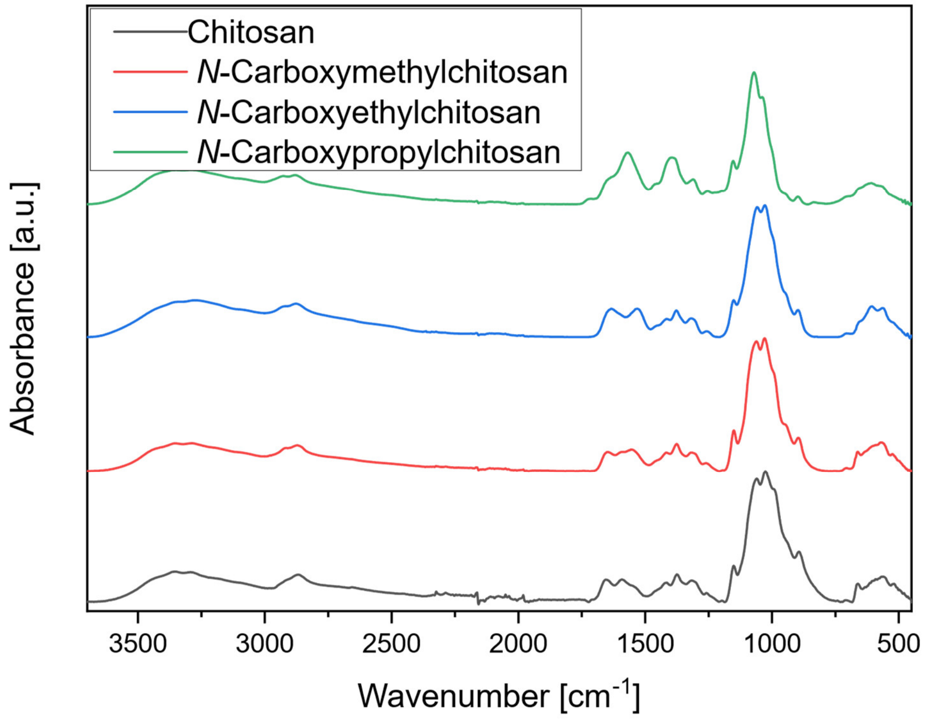


**Figure S-2:** IR-spectra of *N*-carboxyalkyl chitosan.

***Table S-2.*** *N-carboxymethylchitosan with different degrees of substitution.*

| equivalents monochloroacetic acid | Degree of substitution / % |
|---|---|
| 0.1 | 10.2 ± 0.6 |
| 0.25 | 25.9 ± 1.4 |
| 0.5 | 49.9 ± 0.8 |
| 1 | 99.3 ± 1.3 |

***Table S-3.*** *Absorption capacity of N-Carboxyethylchitosan depending on the degree of substitution.*

| Equivalents of 3-chloropropionic acid [-] | Degree of substitution [%] | Absorption capacity [$g \cdot g^{-1}$] |
|---|---|---|
| 0.3 | 12.0 ± 0.7 | 10.3 ± 0.5 |
| 0.6 | 21.4 ± 1.1 | 28.7 ± 0.5 |
| 1 | 27.9 ± 1.5 | 45.9 ± 1.2 |
| 2 | 43.0 ± 1.7 | 20.7 ± 1.8 |
| 3 | 56.4 ± 2.2 | 28.8 ± 3.9 |

***Table S-4.*** *Absorption capacity of N-Carboxypropylchitosan depending on the degree of substitution.*

| Equivalents of 4-chlorobutyric acid [-] | Degree of substitution [%] | Absorption capacity [$g \cdot g^{-1}$] |
|---|---|---|
| 0,5 | 6.9 ± 0.3 | 17.7 ± 5.3 |
| 1 | 12.8 ± 0.8 | 24.3 ± 0.8 |
| 2 | 27.6 ± 1.2 | 29.1 ± 3.6 |
| 3 | 46.4 ± 1.5 | 38.4 ± 0.3 |

**References:**


An, N. T., Thien, D. T., Dong, N. T., & Dung, P. L. (2009). Water-soluble N-carboxymethylchitosan derivatives: Preparation, characteristics and its application. *Carbohydrate Polymers,* 75(3), 489-497.

dos Santos, Z. M., Caroni, A. L. P. F., Pereira, M. R., da Silva, D. R., & Fonseca, J. L. C. (2009). Determination of deacetylation degree of chitosan: a comparison between conductometric titration and CHN elemental analysis. *Carbohydrate Research,* 344(18), 2591-2595.

Yamaguchi, R., Arai, Y., Itoh, T., & Hirano, S. (1981). Preparation of partially N-succinylated chitosans and their cross-linked gels. *Carbohydrate Research,* 88(1), 172-175.
